# Leptin reduces in vitro cementoblast mineralization and survival as well as induces PGE2 release by ERK1/2 commitment

**DOI:** 10.1007/s00784-020-03501-3

**Published:** 2020-08-20

**Authors:** G. Ruiz-Heiland, J. W. Yong, J. von Bremen, S. Ruf

**Affiliations:** Department of Orthodontics, University of Giessen, Schlangenzahl 14, 35392, Giessen, Germany

**Keywords:** Cementoblasts, ERK1/2, Leptin

## Abstract

**Objectives:**

Juvenile obesity is a complex clinical condition that is present more and more frequently in the daily orthodontic practice. Over-weighted patients have an impaired bone metabolism, due in part to their increased levels of circulating adipokines. Particularly, leptin has been reported to play a key role in bone physiology. Leptin is ubiquitously present in the body, including blood, saliva, and crevicular fluid. If, and to what extent, it could influence the reaction of cementoblasts during orthodontic-induced forces is yet unknown.

**Material and methods:**

OCCM-30 cementoblasts were cultivated under compressive forces using different concentrations of leptin. The expression of *ObR*, *Runx-2*, *Osteocalcin*, *Rank-L*, *Sost*, *Caspase 3*, *8*, and *9* were analyzed by RT-PCR. Western blots were employed for protein analysis. The ERK1/2 antagonist FR180204 (Calbiochem) was used and cPLA2 activation, PGE2, and cytochrome C release were further evaluated.

**Results:**

In vitro, when compressive forces are applied, leptin promotes ERK1/2 phosphorylation, as well as upregulates PGE2 and caspase 3 and caspase 9 on OCCM cells. Blockade of ERK1/2 impairs leptin-induced PGE2 secretion and reduced caspase 3 and caspase 9 expression.

**Conclusions:**

Leptin influences the physiological effect of compressive forces on cementoblasts, exerting in vitro a pro-inflammatory and pro-apoptotic effect.

**Clinical relevance:**

Our findings indicate that leptin exacerbates the physiological effect of compressive forces on cementoblasts promoting the release of PGE2 and increases the rate of cell apoptosis, and thus, increased levels of leptin may influence the inflammatory response during orthodontically induced tooth movement.

## Introduction

In all age groups, the prevalence of obesity has been increasing worldwide over the last decades. It is well known that obesity is an important risk factor for many chronic diseases (WHO) [1]. Due to the excess of adipose tissue, the number and volume of adipocytes are increased. Adipocytes are a highly active endocrine organ which produces and releases a variety of adipokines, in particular, increased amounts of proinflammtory adipokines, such as leptin, and decreased amounts of anti-inflammatory adipokines, such as adiponectin. This imbalance between pro- and anti-inflammatory adipokines results in a subclinical chronic systemic inflammation status of obese patients [2–4].

Being the first adipokine discovered, leptin has attracted special research interest [5]. In general medicine, higher leptin levels have been associated with several chronic diseases, such as type 2 diabetes, cardiovascular diseases, cancer, and arthritis (WHO) [1]. In dentistry, there has been a special focus on the effects of leptin on the periodontal structures. There is some evidence that higher leptin levels do not only support the initiation and progression of periodontitis but are also associated with compromised healing after periodontal therapy [6]. Thus, nowadays, obesity is considered a major risk factor for periodontal disease [7]. Considering the fact that orthodontic tooth movement requires healthy periodontal structures, it must be questioned whether or not orthodontic therapy of obese individuals possibly bears more risks than treatment of normal weight patients due to the mild chronic systemic inflammation status.

Furthermore, it has been shown that leptin directly regulates osteoclast formation through a reduced production of RANK and RANK-ligand as well as an increase of osteoprotegerin, which results in reduced osteoclastogenesis [8, 9]. This impaired bone metabolism could also influence orthodontic tooth movement. During orthodontic therapy, the root of the tooth is translated within the alveolar socket resulting in a compressed periodontal ligament (PDL) on the one and stretched periodontal fibers on the opposing side. In a healthy surrounding, this results in bone resorption on the compressed and bone apposition on the stretched side. If, however, higher leptin levels cause more inflammation and less periodontal healing in addition to a compromised bone resorption due to less active osteoclasts, the rate of tooth movement and/or possible side effects of orthodontic tooth movement, such as root resorption, might be affected.

In the biphasic theory of orthodontic tooth movement, the osteoclast activity becomes of even higher importance [10]. Here, it is assumed that orthodontic tooth movement occurs in two phases: first, the osteoclast-driven catabolic phase where bone resorption takes place; and second, the anabolic phase in which bone formation takes place [10]. The first phase, initiated by cytokines, is required for the second phase to begin, and the second phase is regulated by osteoclast-osteoblast interactions. This implies that both phases are dependent on the presence of active osteoclasts and could thus be affected by increased leptin levels.

In addition to an altered reaction of bone and periodontal fibers due to higher leptin levels in obese patients, the cementoblast reaction needs to be considered. So far, this part of the periodontium has received little attention in leptin research. Although it is well accepted that the cementoblasts lining the root surface play an important role in preventing root resorption during orthodontic tooth movement, the causes and predisposing factors for orthodontically induced root resorption are not fully understood [11]. Several studies have confirmed the destructive effect of leptin on periodontal fibroblasts [6, 12–14]. Here, it has been shown that higher leptin levels are associated with an upregulation of somatostatin receptor 2, indicating a potential mechanism how adipokines could contribute to periodontal destruction through their proinflammatory characteristics [14]. Furthermore, the same research group elucidated how different regenerative periodontal ligament cell functions were negatively influenced by the presence of leptin [12]. Supplementing the available research on fibroblasts, the present study now aimed to analyze the effect of leptin on cementoblasts in the presence or absence of compressive forces as induced during orthodontic tooth movement.

## Material and methods

### Cell culture

The mouse cementoblast cell line OCCM-30 was kindly provided from Prof. M. Somerman (NIH, NIDCR, Bethesda, Maryland). Cells were obtained from the root surface of the first mandibular molars of osteocalcin-transgenic (OC-Tag) mice. These mice contain the simian virus 40 (SV40) large T-antigen under the control of the osteocalcin promoter, and thus, only cells that express OC also express Tag in accordance with an immortalized cell line [15].

Cell passages 3–5 were cultivated until confluence using α-MEM (11095-080, Gibco) supplemented with 10% FCS (10270-106, Gibco) and 1% penicillin/streptomycin (15140-122, Gibco). To promote calcification, 50 μg/ml ascorbic acid (6288.1, Roth) and 10 mM β-glycerophosphate (#35675, Calbiochem) were added to cell media using different concentrations of mouse leptin (CYT-351, Prospec). Cementoblasts were cultivated under compressive forces as described by Proff et al. [16]. For this purpose, 33-mm diameter glass cylinders with pulled surfaces were fabricated by Reichmann Feinoptik, Brokdorf, Germany. Cylinders of different volumes were fabricated in order to reach pressure forces of 1.2, 2.4, and 4.8 g/cm^2^, respectively.

The cells were seeded into 6-well plates at a density of 3 × 10^4^ cells/well until confluence and covered with the glass cylinders afterwards. Cells were cultivated under compression over different time periods.

### Calcified matrix assessment

Cells, plated on 6 wells/plates, were washed with phosphate buffered saline solution, fixed with 70% ethanol, and further stained during 5 min with Alizarin-Red (A5533, Sigma-Aldrich), pH 4.2. Calcified matrix formation was assessed by inverted phase contrast microscopy (Leica) using the LASV4.8 software (Leica). Calcified nodules were surrounded; thereafter, the covered area was measured as previously described [17]. A total of eight different microscopic areas per well and a minimum of 6 wells per group were evaluated.

### Alkaline phosphatase enzymatic activity

Alkaline phosphatase enzymatic activity was measured according to an established protocol. Briefly, cells were collected in 0.2% Triton-X and homogenized using an ice-cold Dounce grinder. The protein concentration was measured using Pierce^TM^ BCA protein Assay Kit (23225, Thermo Scientific). Alkaline phosphatase enzymatic activity was measured using p-Nitrophenylphosphat solution (P7998, Sigma) pH 10.5 as substrate and 4-nitrophenol solution (N7660, Sigma) diluted in 0.2% Triton-X as a standard. Plates were read at 405 nm (xMark^TM^, Microplate Absorbance Spectrophotometer, 1681150 BioRad). A total of 6 wells per group were evaluated.

### Quantitative mRNA expression assay

RNA was isolated employing RNeasy Mini Kit (Cat. N° 74104, Qiagen) and treated with RNAse-Free DNase Set (Cat. N° 79254, Qiagen). One-microgram RNA was transcribed into cDNA using iScript^TM^ CDNA Synthesis Kit (Cat. N° 170-8891, BioRad) according to the manufacturer protocol.

TaqMan real-time polymerase assays were performed utilizing TaqMan Universal PCR Master Mix (4304437, Applied Biosystem) and the following TaqMan Gene Expression assays: *Bcl-2* (Mm00477631_m1, Applied Biosystems); *Caspase-3* (Mm01195085_m1, Applied Biosystems); *Leptin Receptor* (Mm00440181_m1, Applied Biosystems); *Caspase-9* (Mm00516563_m1, Applied Biosystems); *Rank-L* (Mm00441906_m1, Applied Biosystems); *Runx-2* (Mm00501584_m, Applied Biosystems); *Caspase-8* (Mn00802247_m1, Applied Biosystems); *Cox-2* (Mm03294838_g1); *Pges* (Mm00452105_m1); *Sost* (Mm00470479_m1). Target gene expressions were normalized to the expression of *β-Actin* (Mm00607939_s1, Applied Biosystems) as housekeeping gene. Thresholds were amplified and detected using CFX96TM Real-Time System Cycler (Bio-Rad). Results were analyzed using the Bio-Rad CFX Manager 3.1 software. Each experiment was repeated at least three times.

### Western blot

Cells were collected in RIPA buffer Pierce^TM^ (89901, Thermo Scientific) supplemented with 3% phosphatase and protease inhibitors (78442, Thermo Scientific). Protein concentration was measured using Pierce^TM^ BCA Protein Assay Kit (23225, Thermo Scientific) on a Nanorop 2000 Spectrophotometer (Thermo Scientific). Protein aliquots were separated by electrophoresis on SDS polyacrylamide gels and blotted to a nitrocellulose membrane (1704271, Bio-Rad) using Trans-Blot Turbo Transfer System (Bio-Rad). Ponceau S solution (P7170, Sigma Aldrich) staining was employed to visualize the transferred protein bands.

Membranes were blocked with 5% non-fat milk (T145.1, ROTH) and incubated for 1 h at room temperature employing the following antibodies: leptin receptors (ObR) (ab5593, Abcam); ERK1/2 (MBS8241746, BIOZOL), dilution 1:1000; phospho-ERK1/2 (44-680G, Thermo-Fisher) dilution 1:1000; JNK (MBS840351, BIOZOL) dilution 1:500; phospho-JNK (07-175, Thermo-Fisher) dilution 1:500; P38 MAPK (9212, Cell Signaling Technology) dilution 1:1000; phospho-P38 MAPK alpha (MA5-15182, Thermo-Fisher) dilution 1:500; STAT1 (AHP2527, Bio-Rad) dilution 1:1000; phospho-STAT1 Tyr701 (05-1064, Thermo-Fisher) dilution 1:1000; phospho-STAT1 S727 (ab109461, Abcam) dilution 1:1000; STAT3 (PA1-86605, Thermo-Fisher) dilution 1:1000; phospho-STAT3 Ser727 (OPA1-03007, Thermo-Fisher) dilution 1:500; Cytochrome C (ab65311, Abcam) dilution 1:1000; cPLA2α (orb100010, BIOZOL) dilution 1:1000; cPLA2β (ab198898, Abcam) dilution 1:1000; SHP2 (PA5-27312, Thermo Fisher) dilution 1:1000; COX 2 (ab62331, Thermo-Fisher) dilution 1:1000. As loading control, β-ACTIN (ab8227, Abcam) dilution 1:2000 was employed. The secondary antibodies: Polyclonal Goat Anti-Rabbit (P0448, Dako); Rabbit Anti-Goat (P0160, Dako); and Polyclonal Goat Anti-Mouse (P0447, Dako) Immunoglobulins/HRP at a dilution 1:2000, were used. The membranes were developed utilizing Amersham ECL Western Blotting Detection Reagents (9838243, GE Healthcare) and detected with Amersham Hyperfilm ECL (28906836, GE Healthcare) on OPTIMAX X-Ray Film Processor (11701-9806-3716, PROTEC GmbH).

### Cytosolic phospholipase A2 assay

Prior to the experiment, OCCM-30 cells were cultivated overnight in starvation media: α-MEM (11095-080, Gibco) supplemented with 0.5% FCS (10270-106, Gibco), 1% penicillin/streptomycin (15140-122, Gibco), 50 μg/ml ascorbic acid (Art. 6288.1, Roth), and 10 mM β-glycerophosphate (#35675, Calbiochem). Cells were cultivated either under compression of 2.4 g/cm^2^ or without compression and with and without the addition of 50 ng/ml leptin (CYT-351, Prospec). To evaluate the effect of ERK1/2 activation on cytosolic phospholipase A2 (cPLA2) regulation, the ERK inhibitor II FR180204 (328007, Calbiochem) was employed. The inhibitor FR180204 (0.2 μg/ml) was added to the cell culture 1 h before experiment start. Cells were collected in phosphate buffer (pH 5.8) and sonicated (Branson Sonifier 150). Phospholipase activity was detected using Cytosolic Phospholipase A2 Assay Kit (ab133090, Abcam) according to the manufacturer protocol. Plates were read at 405 and 414 nm (xMark^TM^ Microplate Absorbance Spectrophotometer, 1681150 Bio-Rad).

### Cytochrome C release test

Cells were washed in cold PBS, collected in 100-μl Cytosol Extraction Buffer containing DTT and protease inhibitors (ab65311, Abcam) and immediately homogenized using Branson Sonifier 150 on ice. Protein concentration was measured employing Pierce^TM^ BCA protein Assay Kit (23225, Thermo Scientific) on a Nanodrop 2000 Spectrophotometer (Thermo Scientific). Ten-microgram protein aliquots were loaded on a 12% SDS-PAGE gel, and western blot was performed using the following manufacturer instructions. After membrane develop, the films were scanned (MC332, OKI), and images were analyzed using the Image J 1.42q (National Institutes of Health, USA). Briefly, images were converted to grayscale 8 bit. Around the first lane, a rectangle was drawn using the rectangular selection tool. This first line, corresponding to the control group, served as a reference. Using the function Analyze>Gels>Plots Lanes, the intensity of every other lane in the image was analyzed. Results were expressed as percentage of the total size of all of highlighted peaks. Values were pasted into Excel for further analysis.

### Elisa test

Prostaglandin E2 (PGE2) release was measured on cell supernatants using a highly sensitive PGE2 ELISA Kit (ADI-900-001, Enzo-Lifesciences). Plates were read at 405 nm and results were analyzed with xMark^TM^ Microplate Absorbance Spectrophotometer (1681150, Bio-Rad) software.

### Statistical analysis

The results were analyzed using ANOVA or Mann-Whitney *U* tests. A *p* value ≤ 0.05 was considered significant. For data distribution, the Shapiro-Wilk test and the Kolmogorov-Smirnov test were used. In addition, QQ plots were performed. Statistical analysis was carried out using the GraphPad Prism 8 software.

## Results

### Leptin receptors are expressed on cementoblasts and leptin reduces cell mineralization

**Fig. 1:**
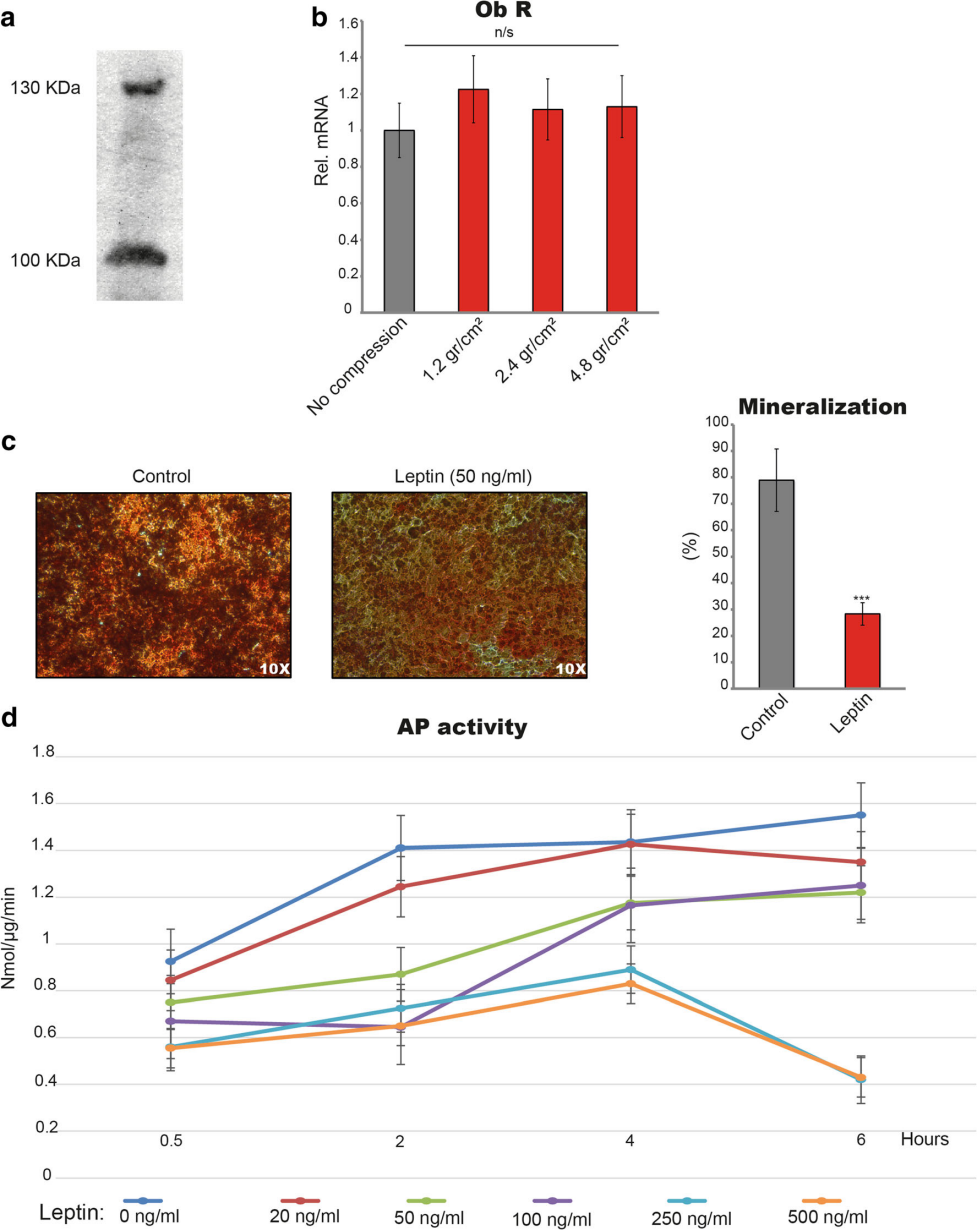
Leptin receptors are present in OCCM-30 mouse cementoblasts. **a** The short isoform α as well as the long isoform β of leptin receptors were detected by WB at 100 kDa and 130 KDa, respectively (Fig. 1a). The application of compressive forces of 1.2 g/cm^2^, 2.4 g/cm^2^, and 4.8 g/cm^2^ did not significantly affect *Ob* receptor expression at mRNA level (Fig. 1b). Leptin added to cell culture reduced mineralized matrix production (***p < 0.001) (*n* = 6). Pictures show alizarin red staining of OCCM cells stimulated during 21 days (Fig. 1c). Leptin reduced alkaline phosphatase enzymatic activity in a dose-dependent manner (Fig. 1d). Each experiment was repeated three times. Bars indicate mean ± SD and stars indicate statistical significance

First, we performed western blot analysis of OCCM cells to determine if and which form isoforms of Ob receptors are present in the cells. The results showed that the long isoform β as well as the short isoform α of leptin receptors is expressed at 130 KDa and 100 KDa, respectively (Fig. 1a). Furthermore, we aimed to elucidate if compression has any influence on the expression of leptin receptors at mRNA level. Therefore, we cultivated cells under compressive forces of 1.2 g/cm^2^, 2.4 g/cm^2^, and 4.8 g/cm^2^ and collected the cells for RNA isolation after 30, 60, 120, 240, and 480 min: Compression did not significantly alter ObR expression at mRNA level at any of the timepoints evaluated (*p* > 0.05) (Fig. 1b).

The sustained stimulation with mice leptin interferes with the normal mineralization of cementoblasts. After 21 days, cells cultivated in the presence of leptin show a markedly impaired mineralization in comparison with control group (28.34 ± 8% vs. 78.93 ± 7.9% of mineralized surface). As expected, leptin reduced AP enzymatic activity and it was observed that this effect occurs dose-dependent. Cells cultivated with adipokine concentrations of 250 ng/ml and 500 ng/ml show similar results after 6-h leptin stimulation, suggesting a saturation dose of leptin on cells (Fig. 1c and d).

### Leptin upregulates COX2 expression as well as STAT1, STAT3, and ERK1/2 phosphorylation irrespective of compression

**Fig. 2:**
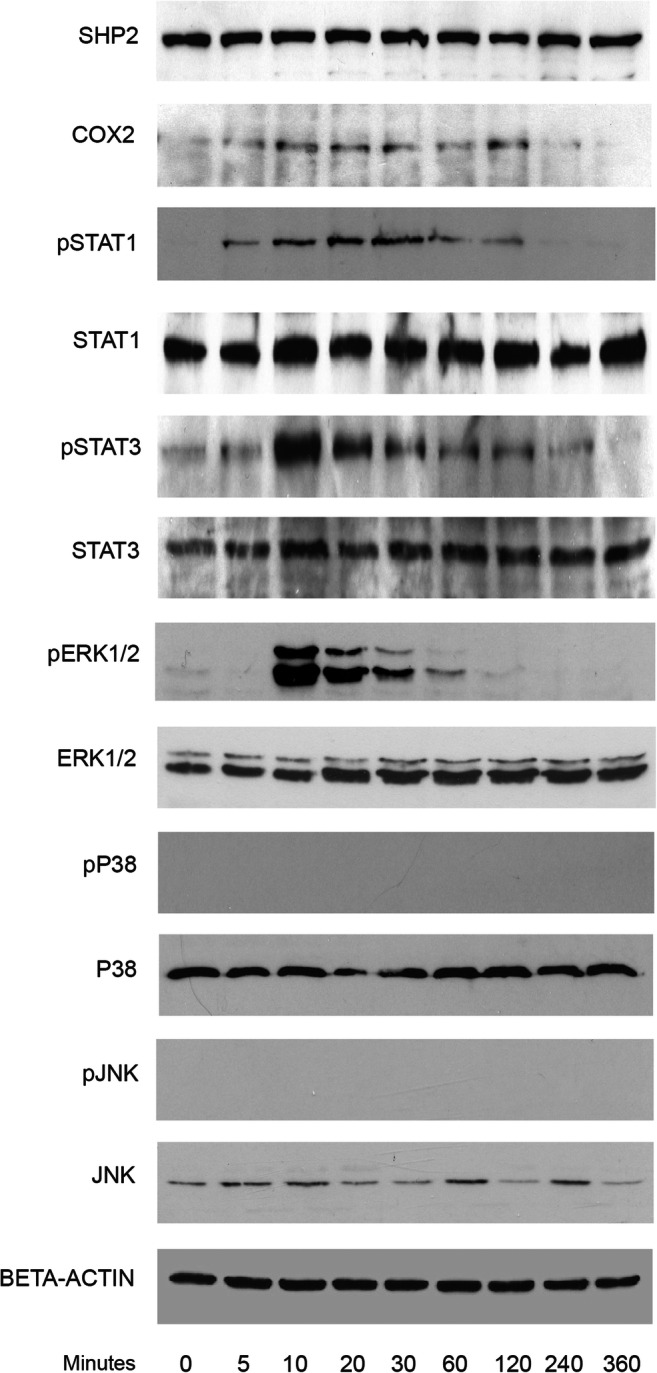
Kinetic analysis performed on OCCM-30 cells after stimulation with leptin. The expression of SHP2; COX2; p-STAT1; STAT1; p-STAT3; STAT3; p-ERK1/2; ERK1/2; p-P38; P38; p-JNK; and JNK were visualized by western blot: Leptin upregulates p-STAT1; p-STAT3; and p-ERK1/2 as well as COX2 on OCCM cells whereas no activation of P38 or JNK was observed. Each experiment was repeated three times

Taking into consideration previous data on signals activated by leptin, we stimulated OCCM cementoblasts with leptin and performed western blot analysis at different timepoints (Fig. 2). Cells stimulated with a saturation dose of leptin (500 ng/ml) show activation of COX2, 10 min after adipokine addition. In the same manner, cells show upregulation of STAT3 phosphorylation whereas STAT1 phosphorylation occurs after 5 min. A decrease on phosphorylation levels occurs 2 h after stimulation.

A strong phosphorylation of ERK1/2 occurs again 10 min after leptin addition whereas no upregulation in the levels of P38 or JNK was observed.

### Enhanced expression of *Rank-L*, *Cox2*, *Pges*, and *Caspases* mRNA expressions by compressive forces after leptin stimulation

**Fig. 3:**
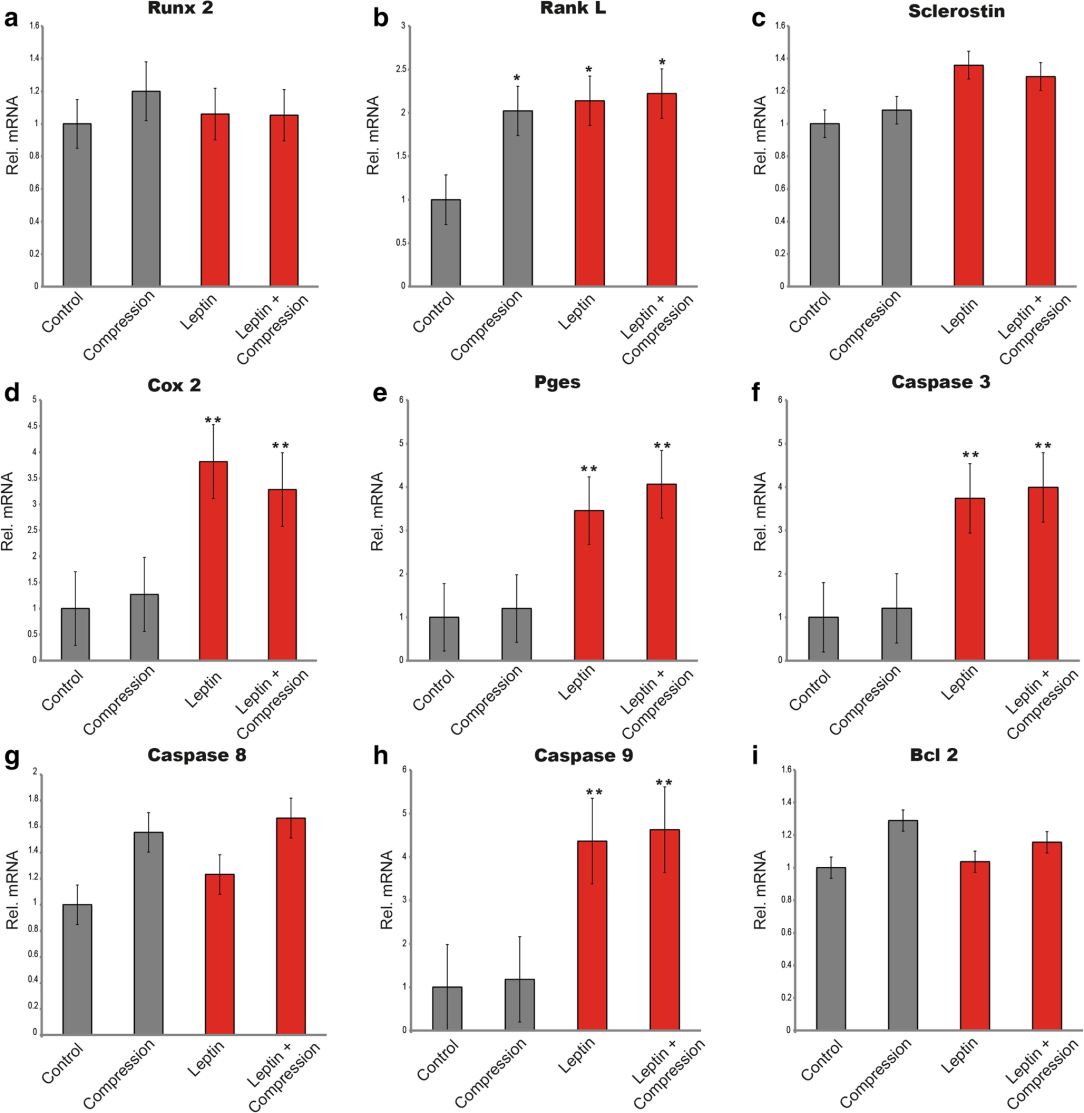
Graphics show the mRNA expression of cementoblasts exposed to compressive forces (2.4 g/cm^2^), with and without co-stimulation with 50 ng/ml leptin (*n* = 3). After 30 min, leptin significantly upregulated *Cox-2*; *PGEs*; *Caspase-3*; and *Caspase-9* expression. The application of compression upregulates *Rank-L* expression irrespective of leptin. Stars indicate statistical significance *p < 0.05 and **p < 0.01. Bars show mean ± SD. Data is normalized to 1

To further elucidate the findings that leptin impairs mineralization, we performed mRNA expression analysis of cells cultivated with and without compression respectively in the presence or absence of leptin, and we analyzed the expression of key molecules involved in cementoblast proliferation, mineralization, and resorption as well as apoptosis (Fig. 3). Cells under compression express increased levels of *Rank-L* irrespective of leptin whereas no significant differences in *Runx-2* levels were observed among groups. The levels of *Sclerostin* mRNA did not vary after compression or leptin stimulation whereas the stimulation with leptin drastically increased the expression of *Cox-2* and *Pge*_*s*_. The expression of *Cox-2* was 2.82 ± 0.55 times higher after leptin stimulation, and *Pge*_*s*_ levels increased to 2.46 ± 0.16-folds. It should be noted that there was hardly any difference between cells stimulated with leptin under compression forces or without compression (*p* ≥ 0.05) both for *Cox-2* and *Pge*_*s*_.

Interestingly, we observed upregulation of *Caspase-3* and *Caspase-9* levels by leptin whereas the levels of *Caspase-8* where not altered after stimulation. On the contrary, the mRNA expression *Bcl-2* remained almost identical among groups.

### Rescue from inflammatory and apoptotic molecules induced by leptin after ERK1/2 blockade

**Fig. 4:**
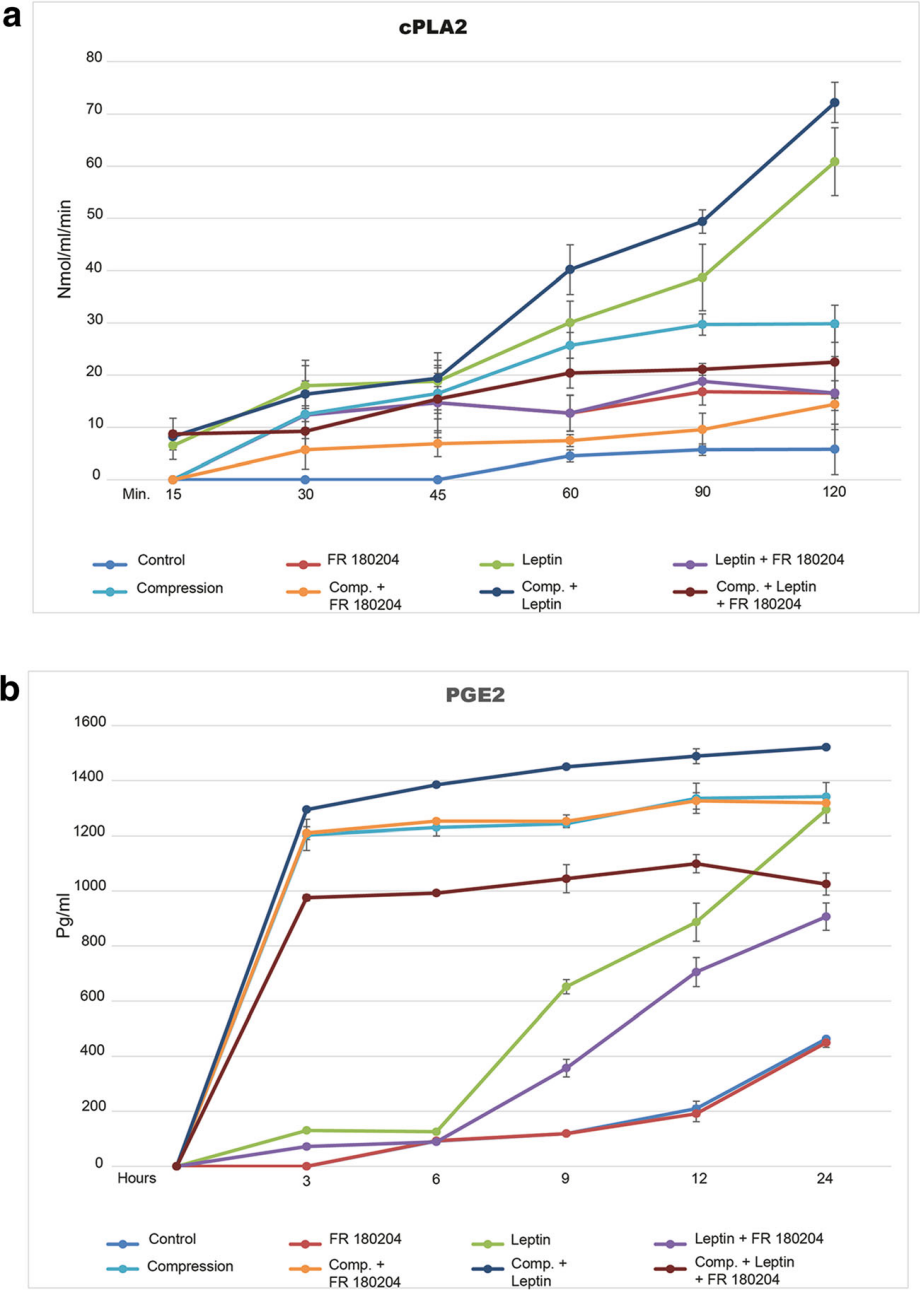
Treatment of cells with FR 180204 for 1 h prior leptin (50 ng/ml) stimulation in the presence or absence of compressive forces has a decreasing effect on cPLA2 activity and PGE2 release. **a**–**b** Leptin activates calcium-dependent cytosolic phospholipase A2 release as well as PGE2 release; both effects were efficiently counteracted by ERK1/2 blockade. Bars indicate mean ± SD

In order to analyze if ERK1/2 plays a central role in cell physiology in response to leptin, we performed a pharmacological inhibition of ERK1/2 using PR180204 (Calbiochem-Merck) and analyzed the cytosolic activity of PLA2 during 2 h: The addition of leptin into cell culture media immediately upregulates cPLA2 activity. After 15 min, the groups stimulated with leptin showed a cPLA2 activity of 6.5 ± 2.65 nmol/min/ml. When compression was applied, the cPLA2 levels increased to 8.2 ± 1.15 nmol/min/ml. After 120 min, these levels continue to increase up to 60.87 ± 6.51 and 72.2 ± 3.83 nmol/min/ml, respectively. The blockade of ERK1/2 significantly reduces cPLA2 activity levels to 16.54 ± 7 nmol/min/ml (*p* = 0.029) and 22.5 ± 6.71 nmol/min/ml (*p* = 0.011). Meanwhile, the cPLA2 levels were undetectable in the control group at almost all timepoints evaluated, reaching a peak after 120 min of 5.78 ± 4.8 nmol/min/ml (Fig. 4a). A total of 2 samples per timepoint (*n* = 2) with 3 measurement per sample were performed resulting in 6 observations per timepoint. Data distribution was analyzed using the Kolmogorov-Smirnov test and visually by normal QQ plots. The experiment was repeated three times based on different samples.

Because it is known that phospholipase A2 drives the regulation of PGE2, we hypothesized that cementoblasts can release PGE2 in response to leptin. As expected, 6 h after leptin addition to cell culture, the PGE2 levels on cell supernatants started to increase to 130.63 ± 2.65 pg/ml and reached 1294 ± 68.51 pg/ml after 24 h. The inhibition of ERK1/2 partially reduced PGE2 release to 906 ± 70 pg/ml (*p* = 0.030) whereas the level in the control group was 463.31 ± 7.48 pg/ml. When compression was applied, the PGE2 level reached 1521.55 ± 93.83 pg/ml after 24 h and 1025.3 ± 56.71 pg/ml with the addition of the ERK1/2 inhibitor (*p* = 0.023). Meanwhile, cells compressed for 24 h under forces of 2.4 g/cm^2^ release PGE2 reaching a peak of 1341.83 ± 73.01 pg/ml and similar levels after ERK1/2 blockade (1318. 8 ± 10.47 pg/ml), suggesting that compression forces can induce PGE2 release on OCCM cells independently of ERK1/2 (Fig. 4b). A total of 2 samples per timepoint (*n* = 2) with 3 measurement per sample were performed resulting in a total of 6 observations per timepoint. Data distribution was analyzed using the Kolmogorov-Smirnov test and visually by normal QQ plots. The experiment was repeated three times based on different samples.

**Fig. 5:**
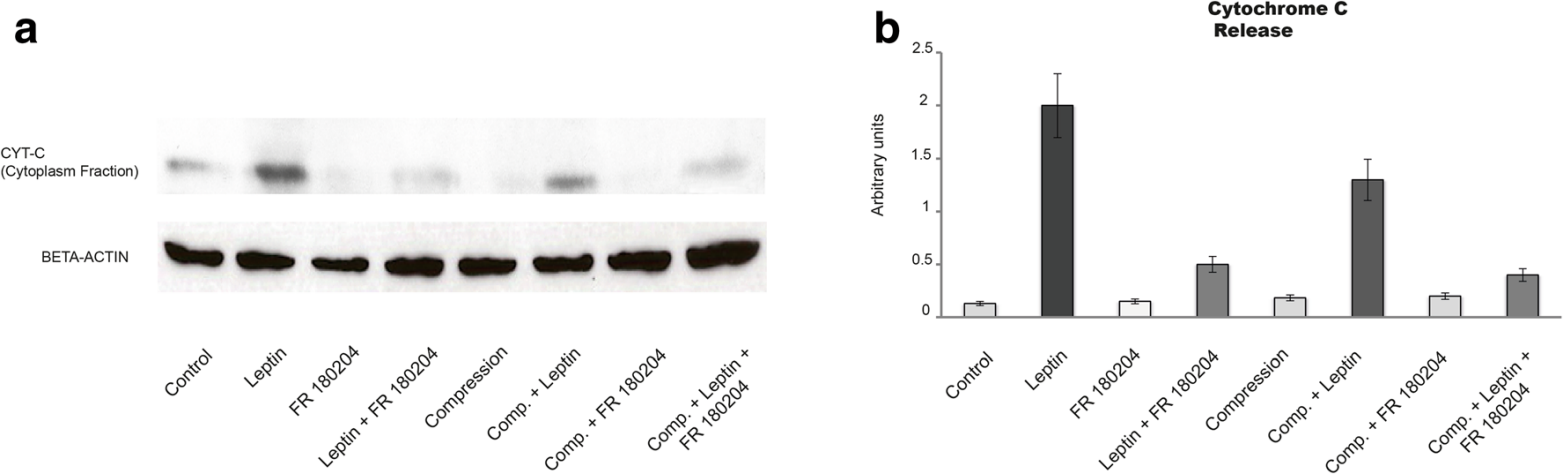
Expression of cytoplasmatic cytochrome C on OCCM cells 4 h after leptin addition (50 ng/ml). Leptin alone or in concomitance with compressive forces upregulates cytochrome C release. Bars indicate mean ± SD

To determine the influence of leptin as inductor of apoptosis on cementoblasts, we analyzed the release of cytochrome C from mitochondria into cytosol by immunoblotting. Four hours after leptin stimulation, OCCM cells show a strong upregulation of cytochrome C release in cells stimulated with leptin and in the group of cells stimulated with leptin and cultivated under compressive forces of 2.4 g/cm^2^. On cells cultivated under compression in the absence of leptin, the release of cytochrome C was remarkably low. The blockade of ERK1/2 impaired its release (Fig. 5a and b).

## Discussion

In the present study, we have shown that cementoblasts can be direct targets of leptin because they express ObR functional receptors. In vitro, leptin enhances the inflammatory response to compressive forces, promoting the upregulation of PGE2, COX2, and RANK-L. We also observed that this adipokine induces cementoblast apoptosis via cPLA2 and cytochrome C release. Leptin strongly activates ERK1/2 and neutralization of ERK1/2 impairs the release of PGE2 as well as cell apoptosis.

To our knowledge, this is the first study to demonstrate that leptin can directly interact with cementoblasts and influence their biological response to compressive forces. Clinical studies have described that the levels of leptin present in crevicular fluid drastically vary during orthodontically induced tooth movement, suggesting that leptin-leptin receptor interactions could play a biological role on this process [18, 19]. Immunohistological analysis performed on primates has shown that leptin receptors are widely expressed in the junctional epithelium and in mineralizing areas of the periodontal ligament [20], as well as it has been demonstrated that the gingiva can directly release soluble leptin receptors into crevicular fluid [21]. Kapur et al. (2010) indicated that leptin receptors are negative modulators of bone mechanosensitivity and that genetic variation in LEPR signaling causes a poor osteogenic response to loading force in mice [22].

Various studies indicate that leptin is an important pro-inflammatory mediator in different tissues of the dentoalveolar system: High levels of this adipokine were detected in infected dental pulps and in periapical granulomas [23], and it is well known that increased levels of leptin in saliva and in crevicular fluids, common in individuals with a high white fat mass, elevate the risk for periodontal disease development [24–26]. In vitro, it has been demonstrated that upregulated levels of leptin promote cytokine release in periodontal ligament cells [24]. Also, the regenerative capacity of cells is impaired in the presence of high levels of leptin due to the downregulation of collagen 1 expression [6].

Regarding the subject that concerns the present study, there is no evidence in vivo that cementocytes play a function in tissue homeostasis, and it has been pointed out that the adjacent periodontal ligament directly influences the physiology of the underlying cement [27, 28]. Nevertheless, in vitro studies indicated that, when cementoblasts are exposed to compressive as well as tensile forces, they can release inflammatory and chemotactic molecules as RANKL and PGE2 [29, 30]. In the present study, we observed that the mRNA expression of *Rank-L*, *Cox-2*, and *Pges* as well as the expression of *Caspase-3* and *Caspase-9* was strongly upregulated by leptin and that this effect was exacerbated when compressive forces were applied. This data let us suppose that increased levels of leptin could be a potential risk for cementoclast activation, especially after the application of compressive forces during orthodontics. However, more clinical evidence, as well as additionally research using primary human cementoblasts, is necessary to further prove the translatability of the results. In the present study, mRNA analysis was performed using *β-Actin* as housekeeping gene. Prior to RT-PCR analysis, the OCCM-30 cells were tested for their expression of *β-Actin* and *GAPDH*, showing a stable and strong expression of both genes with similar results as in primary mouse osteoblasts. However, considering that cementoblasts are part of the conglomerate of dental and periodontal tissues, for future studies, it could be interesting to test the gene expression of PPIB and YWHAZ, as these genes have been shown to be the more stably expressed genes in these tissues [31].

Several studies have described increased levels of circulating leptin in obese individuals [32, 33]. Gröschl et al. 2001 have established a positive correlation between plasma and salivary leptin levels [34]. For obese children, a fourfold increase in salivary leptin levels in comparison with normal weight children has been described (40.4 ng/ml ± 28.8 vs. 9.58 ± 3.1) [35]. During orthodontic tooth movement, the salivary leptin levels in obese individuals may even increase to 89.58 ± 45.4 ng/ml, 1 h after orthodontic forces application, almost doubling the levels measured in lean individuals [36]. At the same time, the rate of orthodontically induced tooth movement was decreased in overweight individuals [36].

**Scheme 1:**
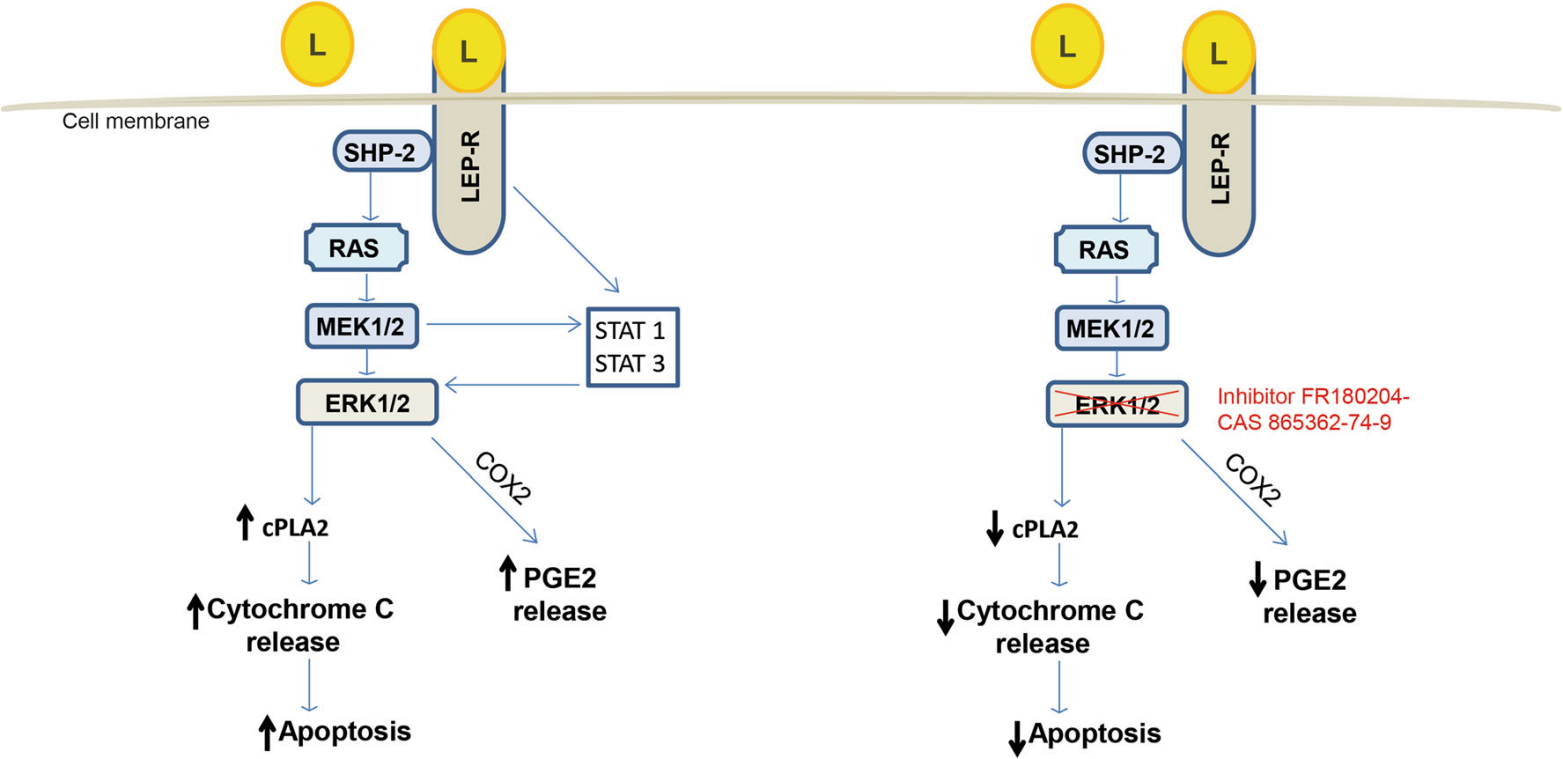
Scheme showing the proposed molecular mechanism induced by leptin on OCCM cells

Previous studies indicate that leptin can induce apoptosis in osteoblast-lineage human bone marrow (hBMSC) cells via the ERK/cPLA2/cytochrome C pathway (Kim, Hong et al. 2003). We found that leptin can interact with cementoblasts in a similar way (Scheme 1), it strongly induces ERK1/2 phosphorylation, even at lower concentrations that mimic biological levels of leptin in saliva (20 to 50 ng/ml) [35, 37].

In accordance with the data shown by Kim el al. (2002) in their experiment using hBMSC, we did not observe phosphorylation of P38 or JNK induced by leptin on cementoblasts [38]. However, we observed that leptin strongly induces STAT3 as well as STAT1 phosphorylation on OCCM-30 cells.

Furthermore, it was observed that exogenous leptin added to the cells promotes PGE2 release as well as cell apoptosis. Both effects are probably regulated by ERK1/2 commitment, as the pharmacological blockade of ERK1/2 seems to have a protective effect on cementoblasts against apoptosis reducing cPLA2 and the release of cytochrome C from the mitochondria. At the same time, the secretion of PGE2 was efficiently counteracted by ERK1/2 blockade. Depending upon the cell type or stimulus, activated ERK1/2 catalyzes several cytoplasmatic and nuclear substrates including regulatory molecules and transcription factors that regulate several cell processes including differentiation, migration, adhesion, survival, inflammation, or even cell apoptosis [39–41]. Hoshi et al. (2014) showed that compressive forces induce osteocyte apoptosis through the ERK1/2 pathway [42]. Another study indicated that ERK1/2 was also activated on MC3T3 osteoblasts after peroxide-induced cell death upstreaming the mitochondria-dependent pathway to initiate the apoptotic signal [43]. Activation of ERK1/2 has been described to promote apoptosis throughout caspase 3 on renal epithelial cells [44]. In addition, animal studies demonstrated that ERK1/2 is activated after ischemic and septic renal injury, proposing that this molecule can activate STAT3 [45]. In vitro experiments using liver cancer cells underline that STAT3 regulates cell survival and that inhibition of this molecule blocks the anti-apoptotic activity of IL-6 on these cells [46]. Interestingly, Kim et al. (2002) showed that STAT1 but not STAT3 was activated by leptin on hBMSC and that STAT1 can directly interact with MEK to induce apoptosis [38]. Other studies using human osteoblasts have shown that phosphorylated STAT1, induced by dexamethasone, decreases the expression of BCL2 and increases in cytochrome C release as well as activates caspase 9 and caspase 3 [47]. In vivo, this mechanism has been demonstrated to be responsible for the progression of steroid-induced avascular necrosis of femoral heads on rats [47]. Other studies using transgenic animals demonstrated that STAT1 deletion can rescue from FGF-induced chondrocyte apoptosis [48].

Our results indicate that the release of PGE2 induced by leptin was partially counteracted by ERK1/2 inhibition. On periodontal cells, stimulation with IL-1 upregulates COX2 that further drives the release of PGE2, and that, its expression can be counteracted by ERK inhibition [49]. Upregulated PGE2 further promotes the release of RANKL and the TRAP^+^ cell formation [49, 50]. Other in vitro studies indicate that leptin alone or in the presence of IL-1 induces COX2 and PGE2 by mitogen-activated protein kinase (MAPK) pathway activation in osteoarthritis cartilage [51]. The COX2-PGE2 augmentation induced by long-term leptin administration has been described to exert a pro-apoptotic effect on renal tubular cells dose-dependently via caspase 3 commitment [52]. One study performed using OCCM cementoblasts revealed that cells exposed to compressive forces can strongly release PGE2 and that this effect can be effectively counteracted using a COX2 inhibitor. In addition, it was observed that high levels of exogenous PGE2 added to OCCM cultures completely abolish mineralization [29]. When we performed the analysis of PGE2 release after the application of compressive forces in the presence or absence of leptin, we observed that the pretreatment of cells with an ERK1/2 antagonist can only partially counteract the release of PGE2 on cells exposed to compressive forces, suggesting that additional factors, as for example, oxygen reduction during compression, could play a role. Kaidi et al. (2006) have demonstrated that hypoxia-inducible factor (HIF-1) directly binds the COX2 promoter in carcinoma cell lines causing PGE2 upregulation. This mechanism facilitates the adaptation to cellular stress imposed by hypoxia [53].

In summary, we conclude that in vitro leptin exerts a deleterious effect on cementoblasts, increasing the release of PGE2 as well as the rate of apoptosis, particularly in cells exposed to compression. The blockade of ERK1/2 partially reverses these processes.
